# Optimization of the Accelerated Solvent Extraction of Caffeoylquinic Acids from Forced Chicory Roots and Antioxidant Activity of the Resulting Extracts

**DOI:** 10.3390/foods11203214

**Published:** 2022-10-14

**Authors:** Etienne Diemer, Morad Chadni, Nabil Grimi, Irina Ioannou

**Affiliations:** 1 URD Agro-Biotechnologie Industrielles (ABI), Centre Européen de Biotechnologie et Bioéconomie (CEBB), AgroParisTech, CEDEX, 51110 Pomacle, France; 2 Transformations Intégrées de la Matière Renouvelable (TIMR), Centre de Recherche Royallieu—CS 60319, ESCOM, Université de Technologie de Compiègne, CEDEX, 60203 Compiègne, France

**Keywords:** forced chicory roots, accelerated solvent extraction, optimization, chlorogenic acid, antioxidant activity, response surface methodology

## Abstract

Forced chicory roots (FCR) are the main but also the least valued by-products of Belgian endive culture. However, they contain molecules of interest for industry such as caffeoylquinic acids (CQAs). This study aims to investigate accelerated solvent extraction (ASE) as a green technique to recover chlorogenic acid (5-CQA) and 3,5-dicaffeoylquinic acid (3,5-diCQA), the main CQAs. A D-optimal design was used to determine the influence of temperature and ethanol percentage on their extraction. Optimal extraction conditions were determined using response surface methodology (RSM) and allow the recovery of 4.95 ± 0.48 mg/g_DM_ of 5-CQA at 107 °C, 46% of ethanol and 5.41 ± 0.79 mg/g_DM_ of 3,5-diCQA at 95 °C, 57% of ethanol. The antioxidant activity of the extracts was also optimized by RSM. The highest antioxidant activity was achieved at 115 °C with 40% ethanol (more than 22mgTrolox/g_DM_). Finally, correlation between the antioxidant activity and the amount of CQAs was determined. FCR can be a great source of bioactive compounds with potential use as biobased antioxidant.

## 1. Introduction

**Figure 1:**
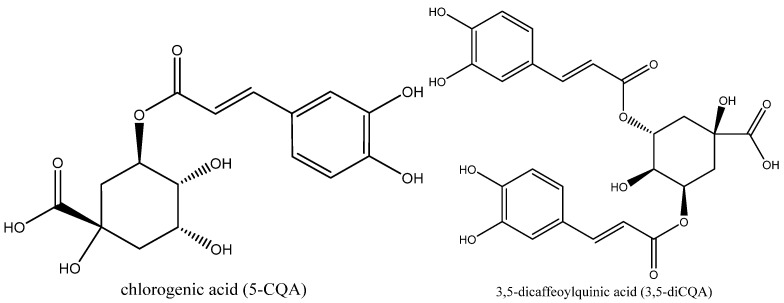
Chlorogenic acid (5-CQA) and 3,5-dicaffeoylquinic acid (3,5-diCQA)**.**

The species *Cichorium intybus* has been of great interest in the agro-industrial economy for several centuries. On the one hand, chicory roots (sativum variety) have had a strongly growing market when used as a substitute for coffee. On the other hand, Belgian chicory (foliusum variety), consumed mainly as salads, is an integral part of the culinary culture of Northern France and Belgium. Moreover, there has been a strong interest in the species *Cichorium intybus* for the extraction and valorization of metabolites composing the chicory roots such as inulin, since the end of the 20th century [1]. Unlike chicory, Belgian endive roots after forcing are the by-products of the endive production and only have low value-added applications as compost or animal feed [2]. However, these forced roots are rich in bioactive compounds with interesting properties such as phenolic compounds and sesquiterpenes lactones [3]. The main phenolic compounds in forced roots are chlorogenic acid (5-CQA) and 3,5-dicaffeoylquinic acid (3,5-diCQA) (Figure 1) [4].

Those caffeoylquinic acids (CQAs) can also be found in many plants and vegetables such as coffee beans, sweet potato, artichokes and *Eucommia ulmoides* [5,6,7]. 5-CQA and 3,5-diCQA are composed of a quinic acid core esterified with one (5-CQA) or two (3,5-diCQA) caffeic acids. These phenolic compounds are biologically active and have high antioxidant, antibacterial and anti-inflammatory activities that can modulate lipid metabolism [8,9,10]. Thus, the recovery of CQAs from forced roots would provide access to high value-added active compounds. Usually, the extraction of CQAs from chicory roots was performed by conventional solvent extraction with water [3] or organic solvent such as ethyl ether, ethyl acetate or a water ethanol-mixture [11,12]. Water is environmentally friendly and less expensive than organic solvents, although it results in a low extraction yield at room temperature [13]. Extraction temperature plays an important role in improving the extraction yield of CQAs [4,14]. Increasing the temperature reduces solvent viscosity and improves the solubility of extracted molecules [15]. To intensify the extraction of CQAs, new technologies have also been used with chicory by-products [16]. Microwave- and/or ultrasound-assisted extraction helps to reduce solvent amount and energy consumption compared to conventional extraction [11,13,14,17]. However, to the best of our knowledge, accelerated solvent extraction (ASE), a promising technology, has not been yet used for chicory root extraction. ASE is a liquid/solid extraction under pressure (100 bar during the whole experiment) in an inert atmosphere (N_2_). These conditions allow the use of temperatures higher than the ebullition point of the solvent. ASE is also suitable to extract bioactive compounds sensitive to oxygen [18,19]. In the literature, no specific optimization of the extraction of 5-CQA and 3,5-diCQA from chicory roots has been carried out with ASE. Existing studies worked at limited temperature (maximum at the boiling temperature) and without a controlled atmosphere (ambient air). ASE makes it possible to study the behaviour of CQAs at subcritical temperature thanks to the pressure in the extraction cells. Reactions at high temperature between extracted compounds and O_2_ are also avoided by using N_2_ in cells. After extraction, the amounts of phenolic compounds are quantified. Most studies in the literature use Folin’s method to quantify these compounds, for convenience [11,14,17]. However, since the HPLC analysis has the advantage of being more accurate and specific than the Folin’s method, the content of 5-CQA and 3,5-diCQA will be determined, here, by HPLC.

The aim of this study was to optimize the extraction of CQAs from forced chicory roots, as well as the antioxidant activity of the extracts, using Response Surface Methodology. With ASE, the effect of the ethanol fraction and temperature were modelled. In addition, DPPH assays were carried out on extracts and standard solutions of CQAs to measure the antioxidant activity. Assumptions were proposed to explain the differences obtained between model solutions and the real extract.

## 2. Materials and Methods

### 2.1. Raw Materials

**Figure 2:**
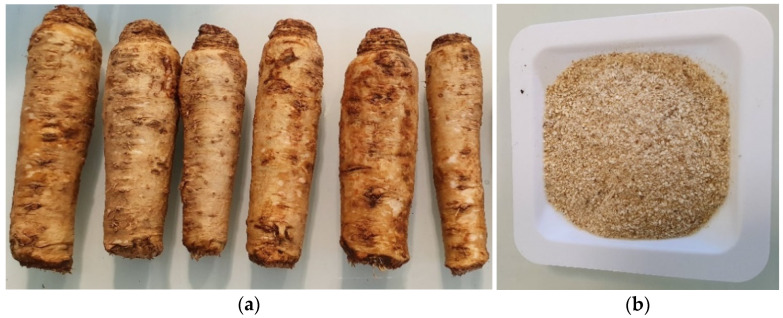
(**a**) Washed forced chicory roots; (**b**) Ground forced chicory roots.

Forced Witloof Chicory roots (FCR) (*Cichorium intybus L*.) of the cultivar “Flexine-Vilmorin” were provided by APEF (l’Association des Producteurs d’Endive de France, Arras, France) experimental station. FCR are the main by-products of the endive cultivation, produced after the forcing stage. FCR were washed with cold tap water (Figure 2a), then cut in slices and dried at 40 °C during 3 days. The dried slices were ground using a commercial blender (WARING, VWR, France) and sieved. The ground material below 500 µm was used for extraction (Figure 2b).

### 2.2. Analytical Reagents and Chemicals

Ethanol (99.9%), acetonitrile (99.9%), formic acid (98–100%) and diatomaceous earth were purchased from Thermo Fisher, (Illkirch, France). Methanol (99%) was obtained from VWR (Fontenay-sous-Bois, France). Ultra-pure water was obtained from a Milli-Q system (Millipore Corporation, USA). 6-hydroxy-2,5,7,8-tetra-methylchromate-2-carboxylic acid (Trolox) and 2,2-diphenyl-1-picryhydrazyl (DPPH), chlorogenic acid standard (>98%) and caffeic acid (CA) (>99%) were purchased from Sigma (Saint-Quentin-Fallavier, France) and 3,4-di-O-caffeoylquinic acid (3,4-diCQA), 3,5-di-O-caffeoylquinic acid and 4,5-di-O-caffeoylquinic acid (4,5-diCQA) (all >90%) were purchased from Carbosynth (Compton, United Kingdom).

### 2.3. Optimization of the Extraction of CQAs

#### 2.3.1. Extraction Protocol

Extractions were performed with an accelerated solvent extraction system (Thermo, Waltham Fisher scientific, USA) (, equipped with a solvent controller unit (Dionex Corporation, Sunnyvale, CA, USA). The extractions were carried out at five different extraction temperatures (40, 65, 90, 115 and 140 °C) and different ethanol percentages (0, 50 and 100%) according to the design of experiments. A fixed mass of 1.10 g (equivalent to 1 g of dry matter (DM)) of powder and 1.10 g of diatomaceous earth were added to a 100 mL ASE container. The cell was filled with roughly 100 mL of solvent, and the exact volume was determined at the end of the extraction with a graduated test tube. Before the beginning of each experiment, the extraction cell was preheated (5 min for an extraction temperature of 40 and 65 °C, 6 min at 90 °C and 115 °C and 7 min at 140 °C). The temperature of the solvent was regulated directly by the system according to the set point. The extraction time was set at 30 min to reach equilibrium. The contents of 5-CQA and 3,5-diCQA were measured by HPLC.

For sugar and protein determination, water extractions were performed to avoid interference between the enzyme and ethanol. Briefly, 1g_DM_ of forced chicory root powder was added to 100 mL of water in a round-bottom flask. The water was previously heated up to 70 °C. The extraction took 30 min and used shaking with a magnetic stirrer.

#### 2.3.2. Response Surface Methodology

A D-optimal design was performed to optimize the extraction process of 5-CQA and 3,5-diCQA with 13 experiments including a triplicate at the central point (Table 1).

The two independent variables in this work are the extraction temperature X1 with different levels (40, 65, 90, 115, 140 °C) and the ethanol fraction into solvent mixture X2 (0, 50, 100% *v*/*v*).

Three responses were modelled: the contents of 5-CQA and 3,5-diCQA as well as the antioxidant activity of the extracts. A second-order polynomial equation was used to mathematically represent responses as a function of the inputs (1):

(1) $$Y_{p}=\;\alpha \;+\sum_{i=1}^{2}\beta_{i}\;X_{i}+\sum_{i=1}^{2}\beta_{ii}\;X_{i²}+\sum \;\sum_{i<j=1}^{2}\beta_{ij}\;X_{i}X_{j}+\;\varepsilon \;$$

where $Y_{p}$ represents the responses (Y _5-CQA_, Y _3.5-diCQA_ and Y _DPPH_), $X_{i}$ and $X_{j}$ are the independent variables, α is a constant and $\beta_{i}$, $\beta_{ij}$, $\beta_{ii}$ are the coefficients of the linear, interaction and quadratic terms. ɛ are variations between observed and predicted values.

The responses are defined as follows:

Y _5-CQA_ = C _5-CQA_ × V/m (2)

where C _5-CQA_ is the concentration of 5-CQA measured by HPLC, V the solvent volume recovered and m the dry mass of biomass used for the extraction.

The Y _3.5-diCQA_ yield was calculated in the same way. For Y _DPPH_, the antioxidant activity was calculated as described below in (5).

Commercial software MODDE v.12.0 (Umetrics AS, Umeå, Sweden) was used to generate the D-optimal design and analyse the experimental data. The effects of each factor were represented by effect diagrams. The model significance was tested using an analysis of variance (ANOVA). The optimal conditions were calculated by the optimizer software tool based on the Nelder–Mead simplex method. The two optima were carried out in triplicate to check compliance with the model. Model coefficients are considered significant when Student’s t-test yields a *p*-value inferior to 0.05.

### 2.4. FCR Characterization

#### 2.4.1. Moisture Determination

The moisture content of FCR was determined, in triplicate, by a MB-35 moisture balance (Mettler Toledo, Viroflay, France). Around 1g of ground FCR was heated at 160 °C until the weight remained stable. Moisture is calculated as follows (3):

MR = (mi − md)/mi(3)

where mi is the mass of the initial sample, md is the mass of dried sample.

#### 2.4.2. Sugar Determination

Fructans were analyzed using the K-FRUC kit (Megazyme, Wicklow, Ireland). First, the compounds likely to create interference (starch, sucrose, glucose oligomers, glucose and fructose) are hydrolyzed and reduced to sorbitol and mannitol. In a second step, the fructans are hydrolyzed. The resulting glucose and fructose react with 4-hydroxybenzoylhydrazine. The final compound is a colored complex whose absorbance at 410 nm is proportional to the concentration of fructose monomers. The complete protocol is referenced by the supplier (Megazyme, Wicklow, Ireland).

D-glucose, D-fructose, and sucrose in the chicory roots were analyzed by using K-SUFRG test kits (Megazyme, Irland). Glucose is measured after two reactions during which it is phosphorylated to glucose-6-phosphate and then oxidized to gluconate-6-phosphate. The absorbance (Aglu) of this compound at 340 nm is proportional to the glucose concentration. Fructose is phosphorylated (fructose-6-phosphate) at the same time as glucose and can be measured in a second step. The absorbance (Afru) is measured at 340 nm. The difference in absorbance (Aglu-Afru) will give the concentration of fructose. The complete protocol is referenced by the supplier (Megazyme, Wicklow, Ireland).

#### 2.4.3. Protein Determination

In the presence of proteins, the Bradford reagent (SIGMA, France), which contains the Coomassie brilliant blue G-250, colors the sample. The absorbance of the sample is proportional to the protein concentration. An aqueous solution of bovine serum albumin (1 mg/mL) was used to prepare standards in a range from 0.01 to 0.2 mg/mL. Extracts were diluted by two-fold. A 75 μL volume of sample is added to Bradford reagent (1.5 mL). The solution is incubated for 30 min at room temperature, protected from light and then the absorbance is measured at 595 nm.

#### 2.4.4. Ash (Dry Basic) Determination

The ash content of FCR was determined according to the Laboratory Analytical Procedure [20]. Around 1 g of ground forced chicory roots was heated using a crucible in a muffle furnace at 575 °C for 4 h and the residual mass recovered after burning is considered to be the ash. Weight was recorded in triplicate after cooling at room temperature to evaluate the ash content.

### 2.5. HPLC Quantification of the CQAs

Extracts were acidified with one drop of HCl to precipitate proteins and filtered through a 0.20 µm filter (Interchim, Uptidisc PTFE). CQAs were quantified by reversed-phase UHPLC-DAD (Ultimate 3000; Dionex, ThermoFisher) equipped with a DAD 3000 diode array detector. Chromatograms were recorded and processed with Chromeleon 6.8 Software. CQAs were separated by a Halo AQ-C18 (Advanced Materials Technology, Wilmington, USA), heated at 35 °C. Elution was performed using a mobile phase composed of 1% formic acid in water (solvent A) and acetonitrile (solvent B) according to the following gradient: 0–1 min 2% B; 1–5.5 min from 2 to 10% B; 5.5–7 from 10 to 15% B; 7–8.5 min 15% B; 8.5–11 min from 15 to 25% B; 11–13.5 min from 25 to 80% B; 13.5–14 min 80% B and 14–15 min from 80 to 2% B. The flow rate was 0.8 mL/min. Sample injection volume was 1 µL. UV acquisition was carried out at 320 nm, 210 nm, 280 nm and 254 nm. 5-CQA, 3,5-diCQA, 4,5-diCQA, 3,4-diCQA and CA were identified and quantified by comparing their relative retention times with their respective standards.

### 2.6. Antioxidant Activity

#### 2.6.1. Measurement of Antioxidant Activity

The antioxidant activity of FCR extracts was assessed by their ability to scavenge DPPH (2,2-diphenyl-picrylhydrazyl) radicals. A calibration curve was made with Trolox (6-hydroxy-2,5,7,8-tetramethyl-chroman-2-carboxylic acid) from 2.5 to 37.5 mg/L. The extract samples were first diluted from 7.5 to 25 with methanol and then 300 µL of solution was mixed with 900 µL of DPPH solution (46.7 mg/L) prepared with methanol. The determination method has been slightly modified from the method described by Brand-Williams et al. (1995) and was similar to our previous work [4]. Briefly, the scavenging capacity was determined by monitoring the decrease in absorbance (UV-vis spectrophotometer Agilent Technologies Cary 60, Wilmington, DE, USA) at 515 nm after 80 min of reaction at room temperature to fully complete the oxidation reaction. Radical scavenging was calculated according to (4):

Radical Scavenging (%) = 100 × (Abs_0_ − Abs_sample_)/(Abs_0_) (4)

where Abs_0_ is the absorbance of the control (without any extract), Abs_sample_ is the absorbance of the extract with DPPH or Trolox solution with DPPH. Antioxidant activity (Y _DPPH_) was calculated using the calibration curve giving absorbance against Trolox concentrations (5). It was expressed as mg Trolox Equivalent (TE) per g dry matter (mg TE/g_DM_).

Y _DPPH_ (mg TE/g _DM_) = D × C _TE_ × V/m _DM_ (5)

where D is the dilution factor, C_TE_ is the concentration of the Trolox equivalent which corresponds to the percentage of inhibition of the extract, V is the total volume of the extract and m _DM_ is the mass of the sample based on dry matter.

#### 2.6.2. Calculation of Antioxidant Activity

For each extract, a predicted value of antioxidant activity—that does not consider potential synergetic effects—can be calculated by multiplying the content of each molecule quantified by the antioxidant capacity determined on model solutions. The predicted value (6) is as follows:

(6) $$Y_{predicted\;activity}=\left(A_{DPPH\;5-CQA}\times C_{5-CQA}\right)+\left(A_{DPPH\;3,5-diCQA}\times C_{3,5-diCQA}\right)\;+\left(A_{DPPH\;4,5-diCQA}\times C_{4,5-diCQA}\right)+\left(A_{DPPH\;3,4-diCQA}\times C_{3,4-diCQA}\right)+\left(A_{DPPH\;CA}\times C_{CA}\right)$$

Where $Y_{predicted\;activity}$ is the predicted value of the antioxidant activity (in mg_Trolox_/g_DM_), $A_{DPPH\;5-CQA}$, $A_{DPPH\;3,5-diCQA}$, $A_{DPPH\;4,5-diCQA}$, $A_{DPPH\;3,4-diCQA}$ are the DPPH activities of the standard solutions in mgTrolox/gmolecule and $C_{5-CQA}$, $C_{3,5-diCQA}$, $C_{4,5-diCQA}$, $C_{3,4-diCQA}$, $C_{CA}$ are the concentrations of the molecules in the extract in g molecule/g _DM_.

### 2.7. Statistical Analysis

The DPPH measurements and the results of the FCR were performed in triplicate, and the mean values and standard deviations were calculated. For extraction experiments, the D-optimal design includes 3 repetitions of the central point of the space studied. The standard deviation was calculated from these triplicates by MODDE software.

## 3. Results and Discussion

### 3.1. Characterization of Forced Chicory Root

The FCR were characterized by measuring the content of different compounds (Table 2). Sugars and proteins are known to be one of the major compounds found in FCR [3,21].

The residual moisture after drying of the forced chicory root (FCR) is 9.2 ± 0.8%. The soluble protein content of the FCR is 0.64 ± 0.13%. Even after the forced stage, FCR still contains a large amount of sugars and their extraction is the subject of ongoing research [21]. Here, FCR contains 10.18 ± 0.54% of fructan, 15.73 ± 1.07% of sucrose, with 2.52 ± 0.05% of D-glucose and 10.24 ± 0.08% of D-fructose according to Megazyme kits. The quantity of each sugar depends on various factors such as cultivar, root stage (forced or not) and storage conditions [22,23]. The inulin metabolism of chicory roots is unclear so far. Compared to the literature, the inulin content of our FCR is low and the D-glucose content is high. Hydrolysis could convert inulin to D-glucose upon storage [24]. The ash content of FCR is 6.60 ± 0.21%; this result is close to the values found in the literature [3].

### 3.2. Optimization of 5-CQA and 3,5-diCQA Extraction Using RSM

#### 3.2.1. Modelling of the Experimental Data

A D-optimal design was used to quantify the effects of two factors on the extraction of 5-CQA and 3,5-diCQA from FCR. 13 extractions were carried out with a triplicate at the central point. The investigated factors were the extraction temperature X1 with 5 levels (40, 65, 90, 115, 140 °C) and the ethanol fraction in the solvent X2 with 3 levels (0, 50 and 100% *v*/*v*). Two responses were quantified, the 5-CQA yield (Y _5-CQA_ in mg/g_DM_) and the 3,5-diCQA yield (Y _3,5-diCQA_ in mg/g_DM_) (Table 3).

The extraction yield of 5-CQA ranges from 0.23 to 5.08 mg/g_DM_. For 3,5-diCQA, the extraction yield ranges from almost 0 to 6.44 mg/g_DM_ (Table 3). Few studies focus on the chlorogenic acid extraction from FCR. For comparison, two studies found yields between 2 and 6 mg/g_DM_ of chlorogenic acid using respectively methanol and 80% ethanol during 15 min [3]. Only [4] quantified the 3,5-diCQA in the forced chicory roots. The 3,5-diCQA extraction optimum (5.97 ± 0.30 mg/g_DM_) is similar to those presented in our study afterwards (5.41 ± 0.79 mg/g_DM_). From these data, two models were determined. Table 4 shows coefficient values from each model.

For 5-CQA extraction, only the quadratic terms of ethanol fraction and temperature are significant (*p* value < 0.05). For 3,5-diCQA, the ethanol fraction and the quadratic terms of temperature and ethanol fraction are significant (*p* value <0.05). Only the significant coefficients are kept in the reduced models. The determination coefficient and the adjusted one are close to 1, demonstrating that the experimental and predicted values are well correlated. Thus, the models can be considered accurate. The regression models are considered significant at 95% because the ANOVA *p* values are well below 0.05. The condition numbers, which reflect a good orthogonality of the models, are less than 10 and so the orthogonality is respected.

The predictive equation made with the reduced model for 5-CQA yield with unscaled coefficients is described by (7).

(7) $$Y_{5-CQA}=1.7796+0.0806\;X_{1}+0.1037\;X_{2}-0.0009\;X_{2}{}^{2}$$

The predictive equation made with the reduced model for 3,5-diCQA yield with unscaled coefficients is described by (8).

(8) $$\;Y_{3,5-diCQA}=-2.48531+0.092545\;X_{1}+0.127728\;X_{2}-0.000442042\;X_{1}{}^{2}-0.00106041\;X_{2}{}^{2}$$

**Figure 3:**
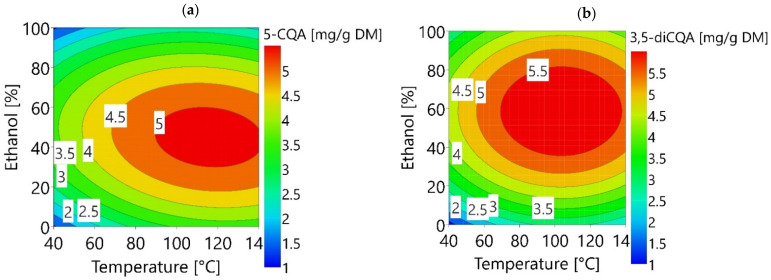
(**a**) Response surface plot of Y _5-CQA_; (**b**) Y _3,5-diCQA._

$Y_{5-CQA}$ and $Y_{3,5-diCQA}$ (mg/g_DM_) can be visualized using response surfaces as a function of temperature and ethanol fraction (Figure 3a,b).

As shown in Figure 3, the yields of 5-CQA and 3,5-diCQA peak near the center of the study domain. Several effects have to be considered to understand the shape of response surface plots. The diffusion of phenolic compounds outside FCR material is not high at relatively low temperatures. The diffusion increases when the solvent heats up, which improves the extraction yields. A second effect appears at relatively high temperature, namely the thermal transformation of phenolic compounds. For 3,5-diCQA, the extraction yield decreases at high temperature, mostly due to the isomerisation of 3,5-diCQA to 4,5-diCQA [4] and 3,4-diCQA (data not shown). For 5-CQA, the decrease in yield occurs at a higher temperature than for 3,5-diCQA. According to Dawidowicz and Typek (2010), 5-CQA degrades to (1S,3R,4R,5R)-5-[3-(3,4-dihydroxyphenyl)-2-hydroxypropanoyl]-1,4,5-trihydroxycyclohexanecarboxylic acid and (1S,3R,4R,5R)-5-[3-(3,4-dihydroxyphenyl)-3-hydroxypropanoyl]-1,4,5-trihydroxycyclohexanecarboxylic acid [25]. The 5-CQA and 3,5-diCQA extraction yields follow a parabolic shape as the ethanol varies. This reflects the importance of solvent polarity on the extraction of CQAs [26]. According to Frosi et al. (2021), ethanol improves the solubility of CQAs and water helps to desorb molecules from the matrix, so an optimum is reached with a mixture of the two. 3,5-diCQA needs a higher ethanol fraction (between 45 to 75%) than 5-CQA (between 30 to 65%) to be well extracted. Dicaffeoylquinic acids are known to be less soluble in water than monocaffeoylquinic acids such as 5-CQA [27]. This can be due to the caffeic acid part, which is slightly soluble in water but quite soluble in organic solvent such as ethanol [28]. With two branched caffeic acids, 3,5-diCQA should be less soluble in water than 5-CQA which has only one.

#### 3.2.2. Validation of the Models on the Optimal Extraction Conditions Predicted

According to the MODDE optimization tool, the optimal conditions for the extraction of 5-CQA are 107 °C and 46% of ethanol. For 3,5-diCQA, the optimal extraction conditions are 95 °C and 57% of ethanol. In these conditions, experiments were carried out in triplicates and compared to the values predicted by the model (Table 5).

According to the Student’s tests, the predicted and observed values are not significantly different (*p* > 0.05). Models at optimal conditions can be considered valid and can be used for prediction.

### 3.3. Optimization of the Antioxidant Capacity of Extracts

#### 3.3.1. Modelling of the Experimental Data

The optimization of the antioxidant activity of the extracts was done using the D-optimal design previously described. The experimental TEAC ranges from 3.41 to 21.42 mgTrolox/g_DM_ (Table 3). These values are in accordance with those obtained in our previous work with FCR [4]. To give an idea of the antioxidant potential of our extracts, a comparison was made with other extracts rich in chlorogenic acid. The TEAC of FCR is twice as high as that of globe artichoke stems [29], three times higher than that of chicory roots var. silvestre [30] and five times more active than apple pomace [31].

From these TEAC data, one model was determined. Table 6 shows coefficient values and statistical parameters. Concerning the significance of the coefficient values shown in Table 6, the quadratic coefficient of the temperature and the interaction term between the fraction of ethanol and the temperature are not significant (*p* > 0.05). The reduced model equation with unscaled coefficients is given below.

(9) $$Y_{DPPH}=-5.5843+0.3424\;X_{1}+0.3986\;X_{2}-0.0038\;X_{2}{}^{2}$$

**Figure 4:**
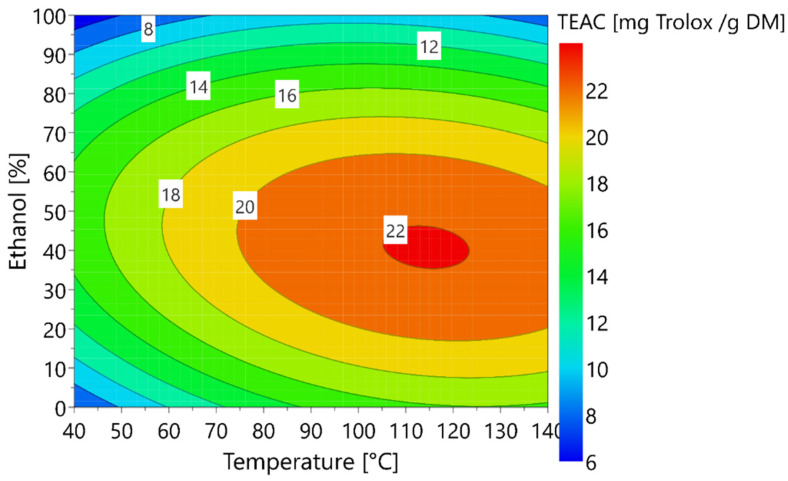
Response surface plot of the TEAC of the extracts.

The TEAC can be represented using surface responses as a function of temperature and ethanol fraction (Figure 4).

The optimal antioxidant activity of the extract is reached using a temperature of 115 °C with 40% ethanol. The optimal operating conditions for maximizing the antioxidant activity do not correspond to those for maximizing the contents of 5-CQA and 3,5-diCQA. This would indicate that the antioxidant activity of the extract is not strictly correlated to the 5-CQA and 3,5-diCQA contents. This could be explained by the presence of other antioxidant molecules in the extract or the existence of synergy between molecules explaining the non-concordance of the two optima.

#### 3.3.2. Validation of the Model

The antioxidant activity was measured for two operating conditions (107 °C with 46% of ethanol and 95 °C with 57% of ethanol). The first corresponds to the optimal extraction condition of 5-CQA and the other is the optimal extraction condition of 3,5-diCQA (Table 7).

The measured and predicted values were statistically compared to each other. According to Student’s tests, the predicted and observed values were not significantly different (*p* > 0.05) for the two conditions. Thus, the model under these conditions is satisfactory and accurate.

#### 3.3.3. Correlation between Antioxidant Activity of Extracts and Caffeoylquinic Acid Contents

According to the HPLC method, five CQAs could be quantified in the extracts: 5-CQA, CA, 3,5-diCQA, 4,5-diCQA and 3,4-diCQA. The last 3 are isomers differing by the position of the two caffeic acids. 5-CQA is the ester of CA (caffeic acid). The antioxidant activity of each CQA was measured using standard solutions (Table 8).

From the TEAC values, there is a relationship between the antioxidant activity of the molecule and its structure. Indeed, the molecules with a single caffeic acid such as CA or 5-CQA have equivalent antioxidant activity. The same observation is made with two caffeic acids. The TEACs of molecules including 2 caffeic acids are twice as high as those containing only one This conclusion can also be found in the literature by comparing the EC50 values of the pure isomers of monoCQAs and diCQAs [10,32].

**Figure 5:**
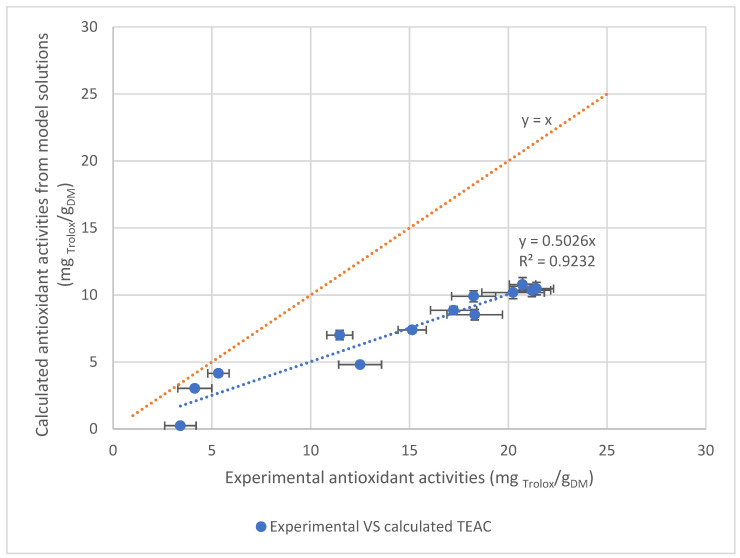
Correlation between calculated and experimental TEAC.

To predict the antioxidant activity of an extract, the TEAC values of each molecule and their proportions in the extract are combined as described in (6). This makes it possible to predict the antioxidant activity of the extract and to compare it to the TEAC measured by DPPH. Figure 5 shows the TEAC calculated as a function of the TEAC measured for each extract of the experimental design.

If the measured TEAC values are perfectly predicted by the calculated values, the points will align with the bisector drawn in orange. However, the points are all below, indicating that the calculation underestimates the antioxidant activity of the extracts.

A linear regression on all the points was carried out. The line, fitting the data with a coefficient of determination of 0.923, is a linear function with a slope equal to 0.5. For example, the TEAC measured for the central point is equal to 21.11 ± 0.36 mgTrolox/g_DM_ whereas the TEAC predicted is equal to 10.48 ± 0.22 mgTrolox/g_DM_. The unexpected 50% of TEAC could be explained either by the presence of other antioxidant molecules or by a synergistic effect between the molecules of the extract.

To explore if a synergistic effect occurs between CQAs or between CQAs and other molecules present in the extract, a model solution with concentrations of CQAs identical those of the central point was prepared and the antioxidant activity measured (Table 9).

This solution has a TEAC of 10.74 ± 0.18 mgTrolox/g_DM_. This value is equivalent to the value calculated by (6) (10.48 ± 0.22 mgTrolox/g_DM_). Thus, the synergistic effect observed above would not be due to the CQAs but would occur between the CQAs and other molecules present in the extract. Some authors have highlighted an increase in TEAC when organic acids such as malic acid or citric acid are present simultaneously with phenolic compounds [33,34]. In addition, organic acids (e.g., malic, citric, shikimic, lactic, quinic, oxalic) have been reported in the composition of chicory biomass [35,36,37]. Moreover, sugars could also modulate DPPH activity. An increase in DPPH activity has been demonstrated with the addition of sucrose in sour cherry puree [38] or in coffee beans during the roasting process [39]. According to the characterization of FCR presented in this paper, a large amount of sugars is present in the extracts and could influence the DPPH activity.

## 4. Conclusions

Extraction of 5-CQA and 3,5-diCQA from FCR can be carried out by ASE. Using response surface methodology, the optimal extraction conditions were found: 107 °C, 46% ethanol to maximize 5-CQA content with 4.95 mg/g_DM_ recovered and 95 °C, 57% ethanol to maximize 3,5-diCQA with 5.41mg/g_DM_ recovered. CQAs exhibit antioxidant activity which can be useful in the cosmetic industry. About 50% of the antioxidant activity measured in the extracts produced can be explained by the presence of caffeoylquinic acids. The remaining 50% comes from the presence of other molecules in the medium, such as sugars and organic acids, either by contributing to the antioxidant activity or synergistically. Further work will need to focus on the identification and on the determination of the molecules contributing to the antioxidant activity in order to adapt the purification process to obtain a concentrate of highly active FCR.

## Figures and Tables

**Table 1:** Experimental design.

**Experiment**	**Temperature X_1_ (°C)**	**Percentage of Ethanol X_2_ (%)**
1	40	0
2	65	0
3	115	0
4	140	0
5	40	50
6	90	50
7	140	50
8	40	100
9	65	100
10	115	100
11	90	50
12	90	50
13	90	50

**Table 2:** Forced chicory root (FCR) characterization.

Moisture Content (g/100 g DM)	9.2 ± 0.8
Ash (g/100 g DM)	6.60 ± 0.21
Proteins (g/100 g DM)	0.64 ± 0.13
Fructan (g/100 g DM)	10.18 ± 0.54
D-Fructose (g/100 g DM)	10.24 ± 0.08
D-glucose (g/100 g DM)	2.52 ± 0.05
Sucrose (g/100 g DM)	15.73 ± 1.07

**Table 3:** Experimental design.

Experiment	Quantity of 5-CQA Extracted (mg/g_DM_)	Quantity of 3,5-diCQA Extracted (mg/g_DM_)	Antioxidant Activity of Extracts (mg_Trolox_/g_DM_)
1	0.23	N.D *	3.41 ± 0.79
2	2.63	2.38	12.50 ± 1.08
3	3.19	2.17	15.14 ± 0.71
4	3.38	2.72	17.23 ± 1.17
5	4.22	5.02	18.30 ± 1.40
6	4.76	5.75	20.24 ± 1.58
7	4.59	4.64	18.24 ± 1.12
8	0.85	2.58	4.14 ± 0.86
9	1.26	3.24	5.33 ± 0.54
10	2.82	4.50	11.47 ± 0.66
11	5.08	5.74	21.20 ± 0.95
12	4.79	6.44	20.72 ± 0.67
13	4.93	5.56	21.42 ± 0.86

* N.D: Not detected.

**Table 4:** 5-CQA and 3,5-diCQA statistical parameters of the model coefficients.

Factors	Coefficients Scaled and Centred	$\mathrm{Coefficients}\;\mathrm{Values}\;\mathrm{for}\;Y_{5-CQA}$	$\mathrm{Coefficient}\;\mathrm{Values}\;\mathrm{for}\;Y_{3,5-diCQA}$
Constant	α	4.9974	5.6817
Temp. (X_1_)	Β_1_	0.8004	0.472837 *
Eth. (X_2_)	Β_2_	−0.3283 *	0.767482
Temp*Temp (X_1_^2^)	Β_11_	−0.8071 *	−1.10511
Eth*Eth (X_2_^2^)	Β_22_	−2.4638	−2.65103
Temp*Eth (X_1_X_2_)	Β_12_	0.3253 *	0.17629 *
Validation parameters			
R^2^		0.928	0.930
R^2^adj		0.877	0.881
Regression (*p* value)		0.001	0.001
Condition number		3.6463	3.6463

* = non significant coefficients.

**Table 5:** Comparison between predicted and experimental values for CQAs extraction.

	5-CQA Yield (mg/g_DM_)	3,5-diCQA Yield (mg/g_DM_)
Optimal conditions for 5-CQA (107 °C, 46% ethanol)		
Predicted values	5.20 ± 0.14	5.64 ± 0.69
Observed values	4.95 ± 0.48	4.94 ± 0.70
p-value (Student test)	0.23	0.14
Optimal conditions for 3,5-diCQA (95 °C, 57% ethanol)		
Predicted values	4.73 ± 0.14	5.77 ± 0.70
Observed values	4.90 ± 0.64	5.41 ± 0.79
p-value (Student test)	0.33	0.30

**Table 6:** DPPH Statistical parameters of the model coefficients.

Factors	Coefficients Scaled and Centred	DPPH Coefficients Values
Constant	α	21.1548
Temp. (X1)	Β1	2.95503
Eth. (X2)	Β2	−2.5831
Temp*Temp (X12)	Β11	−3.40446 *
Eth*Eth (X22)	Β22	−9.54013
Temp*Eth (X1X2)	Β12	−1.90696 *
Validation parameters		
R2		0.916
R2adj		0.856
Regression (p value)		0.006
Condition number		3.64

* Coefficients non significant (*p* > 0.05).

**Table 7:** Comparison between predicted and experimental values for TEAC.

	TEAC (mg_Trolox_/g_DM_)	TEAC (mg_Trolox_/g_DM_)
Optimal conditions	107 °C, 46% ethanol	95 °C, 57% ethanol
Predicted values	21.96 ± 2.68	20.84 ± 2.72
Observed values	24.34 ± 2.46	23.36 ± 3.14
p-value (Student test)	0.16	0.18

**Table 8:** Antioxidant activity of standard solution.

**Molecules**	**TEAC (mg_Trolox_/mmol_molecule_)**
5-CQA	271.7 ± 9.4
3,5-diCQA	508.7 ± 7.1
4,5-diCQA	543.2 ± 4.7
3,4-diCQA	607.8 ± 1.3
CA	283.8 ± 2.8

**Table 9:** Composition of the multi element model solution and real extract.

Compounds	Concentration of Real Extract (mg/g_DM_)	Concentration of Model Solution (mg/g_DM_)
5-CQA	5.28	5.60
3,5-diCQA	5.91	6.41
4,5-diCQA	0.92	1.02
3,4-diCQA	0.34	0.24
CA	0.05	0.06

## Data Availability

Data is contained within the article.
